# Endoplasmic Reticulum Stress Mediates the Anti-Inflammatory Effect of Ethyl Pyruvate in Endothelial Cells

**DOI:** 10.1371/journal.pone.0113983

**Published:** 2014-12-03

**Authors:** Ge Wang, Kan Liu, Yue Li, Wei Yi, Yang Yang, Dajun Zhao, Chongxi Fan, Honggang Yang, Ting Geng, Jianzhou Xing, Yu Zhang, Songtao Tan, Dinghua Yi

**Affiliations:** 1 Department of Cardiovascular Surgery, Xijing Hospital, The Fourth Military Medical University, 127 Changle West Road, Xi′an 710032, China; 2 School of Basic Medical Sciences, The Fourth Military Medical University, 169 Changle West Road, Xi′an 710032, China; 3 Department of Cardiovascular Surgery, Guangdong Provincial Corps Hospital of Chinese People's Armed Police Forces, Guangzhou Medical University, 268 Yanling Road, Guangzhou 510507, China; 4 Department of Air Logistics, The 463rd Hospital of PLA, 46 Xiaoheyan Road, Shenyang 110042, China; 5 Department of Thoracic Surgery, Tangdu Hospital, The Fourth Military Medical University, 1 Xinsi Road, Xi′an 710038, China; Universidade de Sao Paulo, Brazil

## Abstract

Ethyl pyruvate (EP) is a simple aliphatic ester of the metabolic intermediate pyruvate that has been demonstrated to be a potent anti-inflammatory agent in a variety of in vivo and in vitro model systems. However, the protective effects and mechanisms underlying the actions of EP against endothelial cell (EC) inflammatory injury are not fully understood. Previous studies have confirmed that endoplasmic reticulum stress (ERS) plays an important role in regulating the pathological process of EC inflammation. In this study, our aim was to explore the effects of EP on tumor necrosis factor-α (TNF-α)-induced inflammatory injury in human umbilical vein endothelial cells (HUVECs) and to explore the role of ERS in this process. TNF-α treatment not only significantly increased the adhesion of monocytes to HUVECs and inflammatory cytokine (sICAM1, sE-selectin, MCP-1 and IL-8) production in cell culture supernatants but it also increased ICAM and MMP9 protein expression in HUVECs. TNF-α also effectively increased the ERS-related molecules in HUVECs (GRP78, ATF4, caspase12 and p-PERK). EP treatment effectively reversed the effects of the TNF-α-induced adhesion of monocytes on HUVECs, inflammatory cytokines and ERS-related molecules. Furthermore, thapsigargin (THA, an ERS inducer) attenuated the protective effects of EP against TNF-α-induced inflammatory injury and ERS. The PERK siRNA treatment not only inhibited ERS-related molecules but also mimicked the protective effects of EP to decrease TNF-α-induced inflammatory injury. In summary, we have demonstrated for the first time that EP can effectively reduce vascular endothelial inflammation and that this effect at least in part depends on the attenuation of ERS.

## Introduction

The incidence of cardiovascular diseases, such as atherosclerosis (AS), is increasing globally and has become a costly public health issue [1]. The endothelium plays a critical role in the regulation of vascular function and in the development of AS [2]. Increasing evidence suggests that AS is the result of a prolonged and excessive inflammatory process occurring in the vascular wall, often beginning with inflammatory changes to the endothelium and characterized by the expression of adhesion molecules [3], [4]. Multiple cytokines and signaling pathways have been implicated in inflammation-induced vascular endothelial cell (EC) injury [5]. However, the underlying pathophysiological mechanisms of EC inflammatory injury have not been fully elucidated, and more effective treatment methods and drugs to treat EC inflammatory injury need to be explored.

Pyruvate, which is the anionic form of a simple alpha-keto acid, plays a key role in intermediary metabolism as a product of glycolysis and as the starting substrate for the tricarboxylic acid (TCA) cycle [6]. Pyruvate is also an important endogenous scavenger of reactive oxygen species (ROS) and an anti-inflammatory agent [7]. However, its poor stability in solution may limit its use as a therapeutic agent [8]. Ethyl pyruvate (EP), which is a stable and lipophilic derivative of pyruvate, not only overcomes the disadvantages of pyruvate but also possesses many important pharmacological effects [8]. EP can effectively increase the survival rate and/or improve organ dysfunction in animal models of critical diseases, such as severe sepsis, hemorrhagic shock, acute pancreatitis, and acute respiratory distress syndrome, and of intestinal injuries in ischemic models [9]. Notably, EP has been demonstrated to be a potent anti-inflammatory agent in a variety of in vivo and in vitro model systems [10]. However, the protective effects and the mechanisms underlying the action of EP against EC inflammatory injury are not fully understood.

The endoplasmic reticulum (ER) is an organelle involved in protein folding and modification, and it acts as a major intracellular calcium store [11]. ER stress (ERS) is caused by disturbances in the structure and function of the ER and can result from hypoxia, nutrient deprivation, Ca^2+^ imbalances or perturbations in protein glycosylation, leading to the accumulation of unfolded proteins in the ER and the activation of the unfolded protein response (UPR) pathway [12], [13]. The UPR pathway is triggered by three sensors, including activating transcription factor 6 (ATF6), activating transcription factor 4 (ATF4), PKR-like ER kinase (PERK) and inositol-requiring enzyme 1 (IRE1) [13]–[15]. Under normal conditions, these sensors remainin an inactive state, in which they are bound to the chaperone glucose-regulated protein 78 (GRP78). ER stress causes misfolded and unfolded proteins to bind to GRP78, releasing it from the UPR sensors and triggering the UPR by inducing the transcription of genes encoding relevant proteins. This UPR activation thereby reduces global protein synthesis and stimulates ER-associated protein degradation. These activities serve to restore normal ER function, or, when normal ER function cannot be restored, trigger apoptosis [16], [17]. Signaling through the PERK, IRE1, ATF4 and ATF6 pathways can trigger pro-apoptotic signals via the activation of downstream molecules, such as the C/EBP homologous protein (CHOP), the α-subunit of eukaryotic translational initiation factor 2 (eIF2α) and members of the apoptotic family [18]–[20]. Caspase12 is considered to be critical in ERS-induced apoptosis, and it is activated during the ER stress response. Under significantly elevated ERS, the UPR is unable to restore normal cellular function, and signaling switches from pro-survival to pro-apoptotic, in which pro-caspase12 is released, and the apoptotic response is initiated. The released pro-caspase12 is subsequently cleaved to its active caspase12 form, which has been proposed to be a key mediator in the initiation of ERS-induced apoptosis [20]. It is worth noting that the inflammatory response can induce ERS, and the inhibition of ERS can effectively attenuate EC inflammatory injury [21]–[23]. Remarkably, the pharmacological actions of other pyruvate derivatives are closely related to ERS [24]–[26].

Most importantly, we assessed the anti-inflammatory effects of EP in human umbilical vein endothelial cells (HUVECs) and explored the role of ERS in this study.

## Materials and Methods

### Materials

EP, thapsigargin (THA, an ERS inducer), tumor necrosis factor-α (TNF-α), dimethyl sulfoxide (DMSO) and the antibody against TNF-α were purchased from the Sigma-Aldrich Company (St. Louis, MO, USA). The Cell Counting Kit-8 (CCK8) was purchased from Dojindo (Kumamoto, Japan). PERK siRNA and the antibodies against GRP78, intercellular cell adhesion molecule 1 (ICAM-1), matrix metalloproteinase 9 (MMP9) and caspase12 were obtained from Santa Cruz Biotechnology (Santa Cruz, CA, USA). Human soluble adhesion molecule ICAM-1 (sICAM-1), soluble E-selectin (sE-selectin), interleukin (IL-8) and monocyte chemoattractant protein-1 (MCP-1) ELISA kits were obtained from R&D Systems (Minneapolis, MN, USA). The antibodies against phosphorylated-PERK (p-PERK), PERK, ATF4 and β-actin were obtained from Cell Signaling Technology (Beverly, MA, USA). The rabbit anti-goat, goat anti-rabbit and goat anti-mouse secondary antibodies were purchased from the Zhongshan Company (Beijing, China).

### Cell culture and treatments

HUVECs (ATCC, Manassas, VA, USA) were cultured in RPMI 1640 medium (HyClone, UT, USA) supplemented with 10% fetal calf serum, 2 mM L-glutamine, 100 U/ml penicillin, and 100 g/ml streptomycin at 37°C in 5% CO_2_ and 95% air. The EP solution was prepared in DMSO and diluted with culture medium immediately prior to the experiment. DMSO (0.01%) was used as the control group. As described in previous studies [4], TNF-α was used to mimic inflammatory-induced EC injury. The cells were first treated with TNF-α (1, 5 or 10 ng/mL) for 6 h to explore the effects of TNF-α on cell viability, inflammatory cytokines and ERS-related molecules. Further, the cells were treated with EP (1, 5 or 10 mM) for 2 h in the absence or presence of THA (1 µM) and PERK siRNA (pretreated for 24 h). The cells were then treated with TNF-α (10 ng/mL; the concentration was chosen based on preliminary experiments). After the treatments were performed, the cells were harvested for further analysis.

### Analysis of cell viability

CCK-8 was used to measure cell viability. Cells were cultured in a 96-well plate and exposed to various treatments, according to the manufacturer's protocols. The control group was treated with 0.1% DMSO. Then, 10 µl of CCK-8 was added to each well, and the plate was incubated at 37°C for 2 h. Optical density (OD) values were assessed at 450 nm using a microplate reader (SpectraMax 190, Molecular Device, USA), and cell viability was expressed in terms of the OD value.

### Analysis of monocyte adhesion

Monocyte adhesion to ECs was determined using U937 cells (ATCC, Manassas, VA, USA) as previously described by our group [27]. In brief, HUVECs were grown to confluence in 96-well plates. After treatment, the HUVECs were gently washed with serum-free media, and calcein-AM-labeled U937 cells (5×10^4^/ml DMEM medium containing 1% FBS) were then added. After incubation for 1 h, the HUVEC monolayer was gently washed with phosphate-buffered saline (PBS) to remove unbound monocytes. The fluorescence was measured to determine the levels of bound monocytes using a microplate reader (SpectraMax 190, Molecular Device, USA) at excitation and emission wavelengths of 496 and 520 nm, respectively.

### Analysis of sICAM1, sE-selectin, MCP-1 and IL-8 in cell culture supernatants

After treatment, the cell culture supernatants were collected, and the production of sICAM1, sE-selectin, MCP-1 and IL-8 by the HUVECs was measured using special ELISA kits.

### Small interfering RNA transfection

For siRNA transfections, HUVECs were plated onto 6-well, 24-well or 96-well plates and allowed to grow to subconfluency. The cells were transiently transfected with the negative control or PERK siRNA at 100 pM for 24 h using the Lipofectamine RNAiMAX reagent (Invitrogen, Carlsbad, CA, USA) in OPTI-MEM media (Gibco, Carlsbad, CA, USA). The cells were subsequently prepared for use in further experiments.

### Western blotting

After treatment, the HUVECs were lysed in sample buffer (150 mM Tris pH 6.8, 8 M urea, 50 mM DTT, 2% sodium dodecyl sulfate, 15% sucrose, 2 mM EDTA, 0.01% bromophenol blue, 1% protease and phosphatase inhibitor cocktails), sonicated, boiled, run through an 8–12% Bis/Tris gel using 5× MES buffer (Invitrogen) and transferred to an Immobilon NC membrane (Millipore, Billerica, MA, USA). The membranes were blocked with 5% nonfat milk in TBST (150 mM NaCl, 50 mM Tris pH 7.5, 0.1% Tween-20) and then probed with antibodies against GRP78, ICAM1, caspase12 and MMP9 (1∶500) and against p-PERK, TNF-α, ATF4 and β-actin (1∶1000) overnight at 4°C. The membranes were then placed in blocking buffer, washed with TBST, probed with secondary antibodies (1∶5000) in blocking buffer at room temperature for 90 min and washed. Fluorescence was detected using a Bio-Rad imaging system (Bio-Rad, USA), and the signals were quantified using the Image Lab Software (Bio-Rad, USA).

### Statistical analyses

All of the values are presented as the mean ± standard deviation (SD). Group comparisons were performed using ANOVA (SPSS 13.0). All of the groups were analyzed simultaneously using the LSD *t*-test. A difference of *P*<0.05 was considered to be statistically significant. All of the experiments were repeated three times.

## Results

### Effects of TNF-α on cell viability, inflammatory cytokines and ERS-related molecules

**Figure 1:**
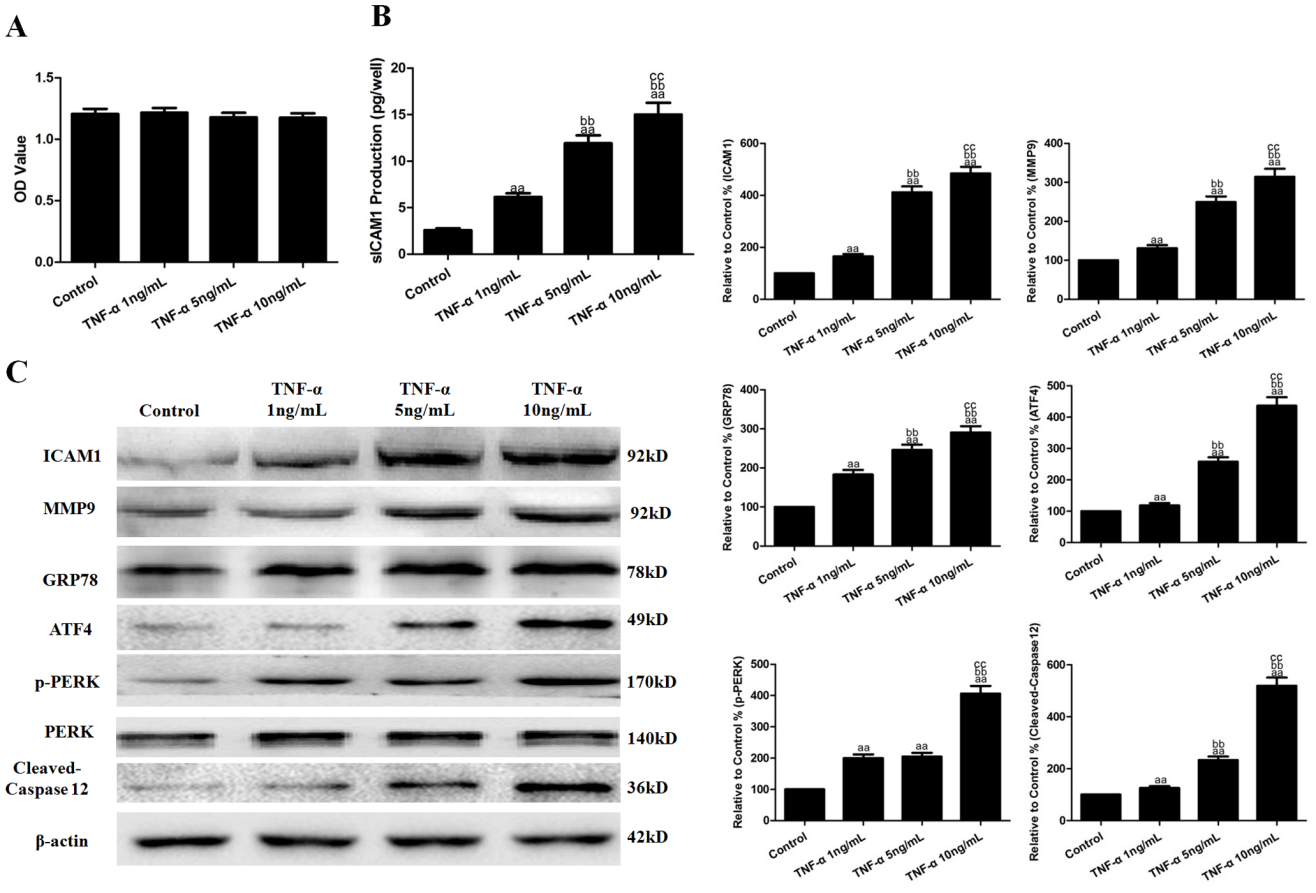
Effects of TNF-α on cell viability, inflammatory cytokines and ERS-related molecules. **A.** Cell viability is expressed as an OD value. **B.** The levels of sICAM1 in cell culture supernatants. **C.** Representative Western blot results showing ICAM1, MMP9, GRP78, ATF4, cleaved-caspase12, p-PERK and PERK protein expression. The results are expressed as the means ±SD, n = 6. ^aa^P<0.01, compared with the control group; ^bb^P<0.01, compared with the 1 ng/mL TNF-α group; ^cc^P<0.01, compared with the 5 ng/mL TNF-α group. EP, ethyl pyruvate; OD, optical density.

HUVECs were first treated with TNF-α (1, 5 or 10 ng/mL) for 6 h to explore the effects of TNF-α on cell viability, inflammatory cytokines and ERS-related molecules. As shown in Fig. 1A, TNF-α treatment had no significant effects on cell viability (vs. the control group, *P*>0.05). However, TNF-α increased sICAM-1 production in the cell culture supernatants to 6.14±0.41, 11.93±0.85 and 15.01±1.26 pg/well, for each respective trial (vs. the control group, *P*<0.01, Fig. 1B). TNF-α also up-regulated ICAM-1 and MMP9 protein expression in the HUVECs (vs. the control group, *P*<0.01, Fig. 1B) in a dose-dependent manner, and the Western blot results are shown in Fig. 1C (vs. the control group, *P*<0.01). In addition, TNF-α increased the expression of the ERS-related protein GRP78, as well as ATF4, cleaved-caspase12 and p-PERK (vs. the control group, *P*<0.01, Fig. 1C). The effects of TNF-α on these proteins were the most obvious at 10 ng/mL, which led to the increased expression of ICAM1, MMP9, GRP78, ATF4, cleaved-caspase12 and p-PERK to 484.09±25.80%, 314.15±20.73%, 290.42±16.46%, 436.48±26.93%, 519.04±31.78% and 406.51±24.18%, respectively. These results suggest that ERS may be involved in EC inflammatory injury. TNF-α at a concentration of 10 ng/mL was selected for further experiments.

### Effects of EP on TNF-α-induced cell viability, monocyte adhesion and inflammatory cytokines

**Figure 2:**
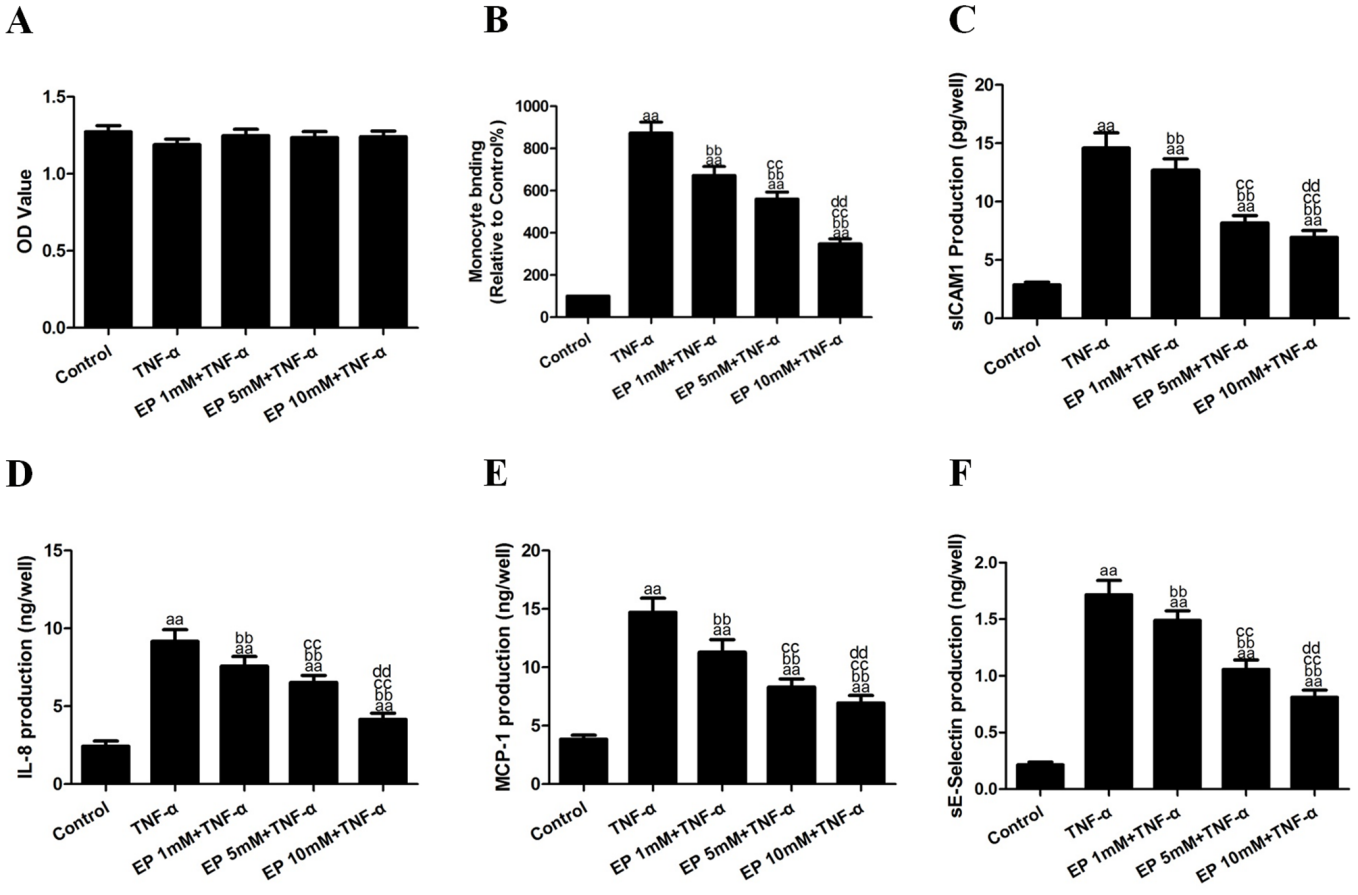
Effects of EP on TNF-α-induced cell viability, monocyte adhesion and inflammatory cytokines in cell culture supernatants. **A.** Cell viability is expressed as an OD value. **B.** The adhesion of U937 monocytes to HUVECs. **C.** The levels of sICAM1 production in cell culture supernatants. **D.** The levels of IL-8 production in cell culture supernatants. **E.** The levels of MCP-1 production in cell culture supernatants. **F.** The levels of sE-selectin production in cell culture supernatants. The results are expressed as the means ± SD, n = 6. ^aa^P<0.01, compared with the control group; ^bb^P<0.01, compared with the TNF-α group; ^cc^P<0.01, compared with the 1 mM EP+TNF-α group; ^dd^P<0.01, compared with the 5 mM EP+TNF-α group. EP, ethyl pyruvate; OD, optical density.

**Figure 3:**
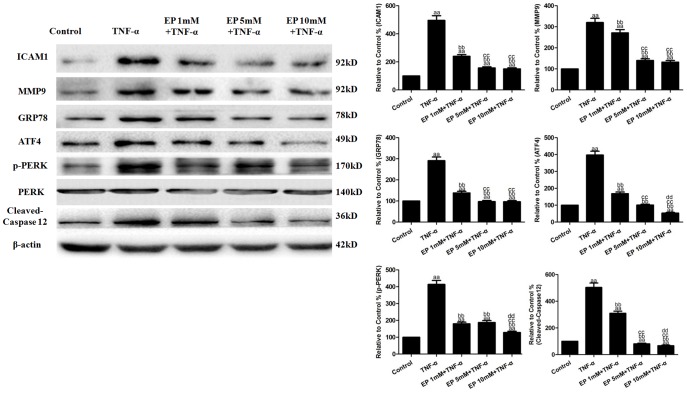
Effects of EP on the TNF-α-induced inflammatory protein and ERS-related molecules in HUVECs. Representative Western blot results showingICAM1, MMP9, GRP78, ATF4, cleaved-caspase12, p-PERK and PERK protein expression. The results are expressed as the means ± SD, n = 6. ^aa^P<0.01, compared with the control group; ^bb^P<0.01, compared with the TNF-α group; ^cc^P<0.01, compared with the 1 mM EP+TNF-α group; ^dd^P<0.01, compared with the 5 mM EP+TNF-α group. EP, ethyl pyruvate.

HUVECs were treated with EP (1, 5 or 10 mM) for 2 h and then subjected to TNF-α(10 ng/mL) treatment. As shown in Fig. 2A, there were no significant differences in cell viability among the groups (*P*>0.05). Exposure of the HUVECs to TNF-α significantly increased their binding to the U937 monocytes to 874.02±51.69% (vs. the control group, *P*<0.01, Fig. 2B). Pretreatment with EP significantly inhibited the TNF-α-induced binding of the U937 monocytes to the HUVECs to 672.17±42.65%, 560.08±34.01% and 348.10±23.84% for each trial, respectively (vs. the TNF-α group, *P*<0.01). In addition, exposure of the HUVECs to TNF-α significantly increased the production of sICAM1, IL-8, MCP-1 and sE-selectin to 14.59±1.30 pg/well, 9.17±0.75 ng/well, 14.71±1.21 ng/well and 1.715±0.128 ng/well in the cell culture supernatants (vs. the control group, *P*<0.01, Figs. 2C–2F), respectively. The EP treatment reversed the TNF-α-induced sICAM1, IL-8, MCP-1 and sE-selectin production (vs. the TNF-α group, *P*<0.01), and the effects were the most obvious at 10 mM EP, which led to decreased sICAM1, IL-8, MCP-1 and sE-selectin production to 6.95±0.57 pg/well, 4.15±0.41 ng/well, 6.94±0.65 ng/well and 0.810±0.064 ng/well. TNF-α (10 ng/mL) significantly up-regulated the expression of the ICAM-1 and MMP9 proteins to 496.15±32.87% and 320.47±19.95% in the HUVECs (vs. the control group, *P*<0.01, Fig. 3). EP treatment reversed the effects of TNF-α, and these effects were the most obvious at10 mM EP, which decreased the ICAM1 and MMP9 expression to 150.23±8.18% and 132.09±8.02% (vs. the TNF-α group, *P*<0.01)

### Effects of EP on the TNF-α-induced ERS-related molecules in the HUVECs

As shown in Fig. 3, TNF-α (10 ng/mL) significantly up-regulated the expression of the GRP78, ATF4, cleaved-caspase12 and p-PERK proteins to 291.16±17.31%, 397.15±22.74%, 503.77±33.60% and 414.06±23.10% in HUVECs (vs. the control group, *P*<0.01). EP treatment reversed the effects of TNF-α, decreasing the GRP78, cleaved-caspase12, ATF4 and p-PERK expression to 96.44±5.50%, 52.94±4.26%, 67.44±4.31% and 128.60±6.88% at 10 mM EP (vs. the TNF-α group, *P*<0.01).

### Effects of EP and THA co-treatment on TNF-α-induced cell viability and inflammatory cytokines

**Figure 4:**
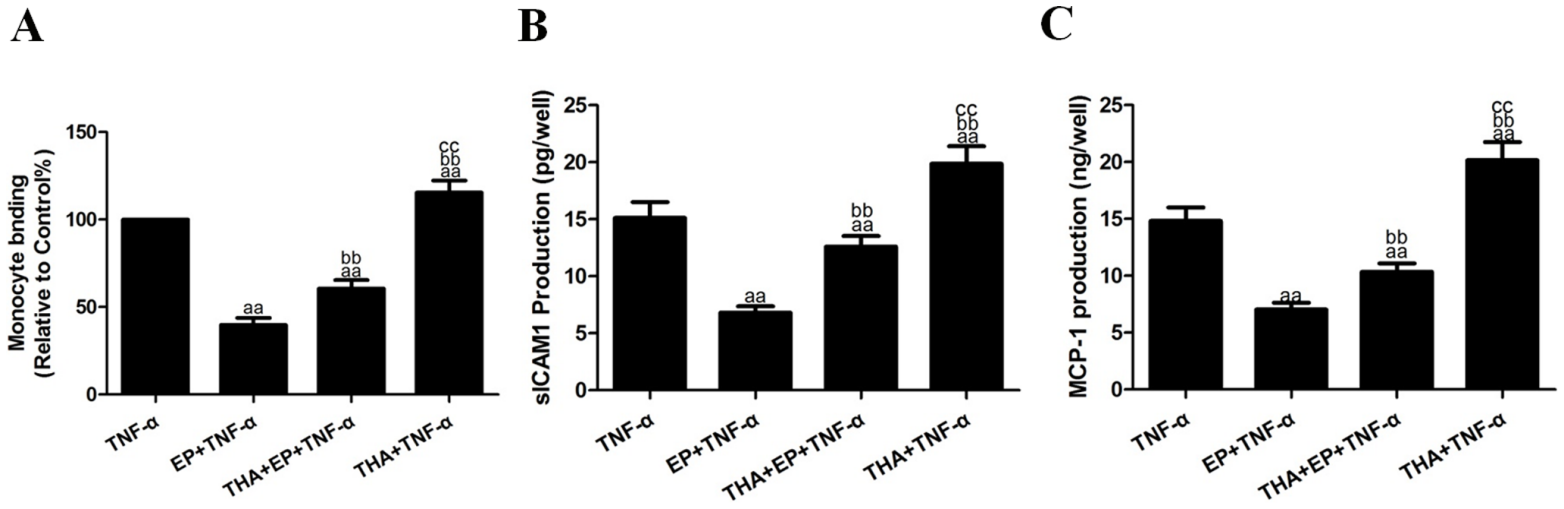
Effects of EP and THA co-treatment on TNF-α-induced inflammatory cytokines in cell culture supernatants. **A.** The adhesion of U937 monocytes to HUVECs. **B.** The levels of sICAM1 production in cell culture supernatants. **C.** The levels of MCP-1 production in cell culture supernatants. The results are expressed as the means ± SD, n = 6. ^aa^P<0.01, compared with the TNF-α group; ^bb^P<0.01, compared with the EP+TNF-α group; ^cc^P<0.01, compared with the EP+THA+TNF-α group. EP, ethyl pyruvate; THA, thapsigargin.

**Figure 5:**
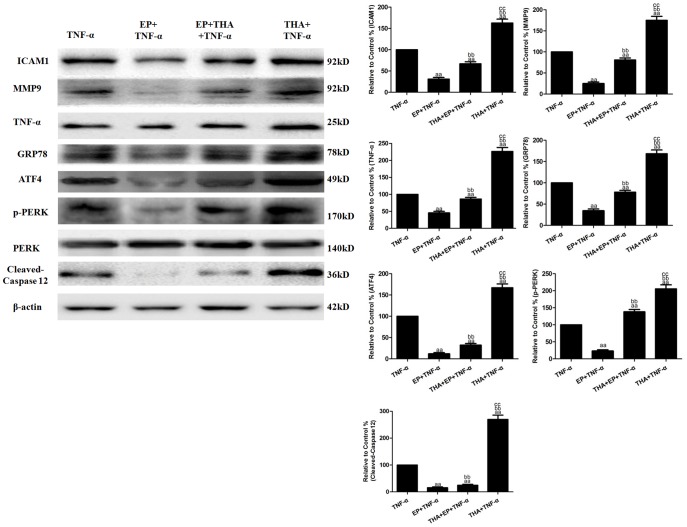
Effects of EP and THA co-treatment on the TNF-α-induced inflammatory protein and ERS-related molecules in HUVECs. Representative Western blot results showing ICAM1, MMP9, TNF-α, GRP78, ATF4, cleaved-caspase12, p-PERK and PERK protein expression. The results are expressed as the means ± SD, n = 6. ^aa^P<0.01; compared with the TNF-α group, ^bb^P<0.01; compared with the EP+TNF-α group; ^cc^P<0.01, compared with the EP+THA+TNF-α group. EP, ethyl pyruvate; THA, thapsigargin.

To explore the role of ERS in the anti-inflammatory effects of EP, HUVECs were treated with EP (10 mM) for 2 h in the absence or presence of THA (1 µM, based on preliminary experiments) and then subjected to TNF-α (10 ng/mL) treatment. EP treatment significantly inhibited the TNF-α-induced binding of U937 monocytes to the HUVECs to 39.81±4.05% and decreased the production of sICAM1 and MCP-1 in the cell culture supernatants to 6.80±0.57 pg/well and 7.03±0.60 ng/well (vs. the TNF-α group, *P*<0.01, Figs. 4A–4C). However, THA and EP co-treatment increased the binding of U937 monocytes to HUVECs to 60.55±4.90% and reversed the down-regulation of sICAM1 and MCP-1 production to 12.59±0.94 pg/well and 10.34±0.75 ng/well (vs. the EP+TNF-α group, *P*<0.01). In addition, EP treatment also decreased ICAM-1, MMP9 and TNF-α protein expression to 31.09±3.80%, 25.14±3.30% and 45.61±4.50% in the HUVECs (vs. the TNF-α group, *P*<0.01, Fig. 5), while THA and EP co-treatment reversed the down-regulation of the expression of the ICAM-1, MMP9 and TNF-α proteins to 67.10±4.65%, 80.95±4.71% and 86.29±5.02% in the HUVECs (vs. the EP+TNF-α group, *P*<0.01). Compared with the TNF-α group, THA+TNF-α treatment not only significantly increased the binding of U937 monocytes to HUVECs and increased sICAM1 and MCP-1 production in the cell culture supernatants (*P*<0.01, Figs. 4A–4C) but also increased the expression of the ICAM-1, MMP9 and TNF-α proteins in the HUVECs (*P*<0.01, Fig. 5).

### Effects of EP and THA co-treatment on the ERS-related molecules in HUVECs

As shown in Fig. 5, EP treatment significantly decreased the expression of the GRP78, ATF4, cleaved-caspase12 and p-PERK proteins to 34.63±4.02%, 11.90±2.68%, 15.69±3.05% and 23.29±3.47% in the HUVECs (vs. the TNF-α group, *P*<0.01), while THA and EP co-treatment reversed the down-regulation of GRP78, ATF4, cleaved-caspase12, and p-PERK protein expression to 78.07±4.35%, 32.29±3.75%, 24.80±3.48% and 138.14±7.02% (vs. the EP+TNF-α group, *P*<0.01). Compared with the TNF-α group, THA+TNF-α treatment significantly increased GRP78, ATF4, cleaved-caspase12 and p-PERK protein expression to 168.42±8.90%, 167.14±9.01%, 269.66±15.89% and 205.60±11.87% in the HUVECs (vs. the TNF-α group, *P*<0.01).

### Effects of PERK siRNA on TNF-α-induced cell viability and inflammatory cytokines

**Figure 6:**
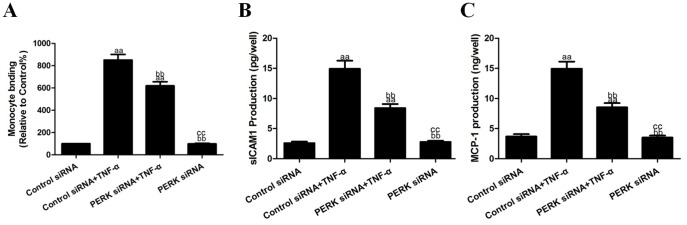
Effects of PERK siRNA on TNF-α-induced inflammatory cytokines in cell culture supernatants. **A.** The adhesion of U937 monocytes to HUVECs. **B.** The levels of sICAM1 production in cell culture supernatants. **C.** The levels of MCP-1 production in cell culture supernatants. The results are expressed as the means ± SD, n = 6. ^aa^P<0.01, compared with the control siRNA group; ^bb^P<0.01, compared with the control siRNA+TNF-α group; ^cc^P<0.01, compared with the PERK siRNA+TNF-α group. EP, ethyl pyruvate.

**Figure 7:**
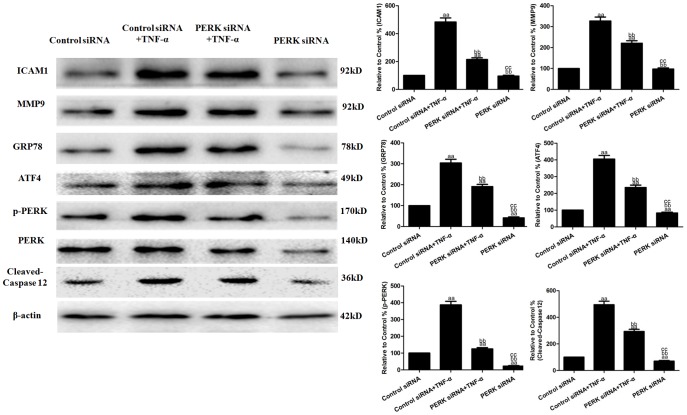
Effects of PERK siRNA on the TNF-α-induced inflammatory protein and ERS-related molecules in HUVECs. Representative Western blot results showingICAM1, MMP9, GRP78, ATF4, cleaved-caspase12, p-PERK and PERK protein expression. The results are expressed as the means ± SD, n = 6. ^aa^P<0.01, compared with the control siRNA group; ^bb^P<0.01, compared with the control siRNA+TNF-α group; ^cc^P<0.01, compared with the PERK siRNA+TNF-α group. EP, ethyl pyruvate.

To further explore the role of ERS in the anti-inflammatory effects, HUVECs were treated with PERK siRNA for 24 h and then subjected to TNF-α (10 ng/mL) treatment. PERK siRNA treatment significantly inhibited the TNF-α-induced binding of U937 monocytes to HUVECs to 619.47±36.92% and decreased sICAM1 and MCP-1 production in the cell culture supernatants to 8.41±0.66 pg/well and 8.52±0.71 ng/well (vs. the control siRNA+TNF-α group, *P*<0.01, Figs. 6A and 6B). In addition, PERK siRNA treatment also decreased ICAM-1 and MMP9 protein expression to 214.01±12.70% and 220.90±11.84% in the HUVECs (vs. the control siRNA+TNF-α group, *P*<0.01, Fig. 7). Compared with the control siRNA group, PERK siRNA treatment alone had no effect on the binding of U937 monocytes to HUVECs and had no effect on sICAM1 and MCP-1 production in the cell culture supernatants (*P*>0.05, Figs. 6A–6B). It also did not affect ICAM-1 orMMP9 protein expression in the HUVECs (*P*>0.05, Fig. 7).

### Effects of PERK siRNA on the TNF-α-induced ERS-related molecules in HUVECs

As shown in Fig. 7, control siRNA+TNF-α treatment significantly increased GRP78, ATF4, cleaved-caspase12 and p-PERK protein expression to 303.90±17.59%, 405.43±21.38%, 494.62±26.05% and 387.62±21.26% in the HUVECs (vs. the control siRNA group, *P*<0.01), while PERK siRNA+TNF-α treatment significantly decreased GRP78, ATF4, cleaved-caspase12 and p-PERK protein expression to 191.28±10.37%, 235.71±14.02%, 293.20±16.07% and 125.09±7.80% (vs. the control siRNA+TNF-α group, *P*<0.01). Compared with the control siRNA group, PERK siRNA treatment alone significantly decreased GRP78 and p-PERK protein expression to 41.52±4.70%, 82.95±5.27%, 70.28±4.81% and 22.13±3.46% (vs. the control siRNA group, *P*<0.01).

## Discussion

Although the pathogenesis of atherosclerotic vascular disease involves multifactorial processes, accumulating evidence demonstrates that inflammation and subsequent endothelial dysfunction play fundamental roles in the initiation and progression of atherosclerosis [28]. Apoptotic endothelial cells contribute to endothelial dysfunction, the destabilization of atherosclerotic plaques, and thrombosis during the initiation and progression of atherosclerosis [29]. Among several proapoptotic factors present in atherosclerotic plaques, oxidized low-density lipoproteins (ox-LDLs) participate in the formation and progression of lesions by triggering lipid storage, local inflammation, TNF-α production, ERS activation, and toxic events, which can cause vascular wall injury and death; plaque transition from stable to vulnerable; erosion and rupture; and subsequent athero-thrombosis [30], [31].

Accumulating evidence suggests that the inflammatory cytokine TNF-α, which is a pleiotropic proinflammatory cytokine, plays an important role in the disruption of vascular function and the subsequent development of vascular disease [32]. Epidemiological studies have demonstrated that TNF-α is remarkably elevated in the plasma and arteries of humans with vascular complications [33]. In addition, TNF-α has been evaluated as an injury-related factor in many studies involving inflammation [4], [27], [28], [32]. Therefore, TNF-α was selected to mimic EC inflammatory injury in this study. Furthermore, TNF-α has been shown to induce the expression and release of a series of adhesion molecules and chemokines involved in the inflammatory response in ECs [34], [35]. Chemokines such as MCP-1 and IL-8 are key mediators in the regulation of enhanced EC-monocyte interactions and subsequent monocyte recruitment to vascular tissues [28]. Cytokines such as TNF-α induce the expression of adhesion molecules on the surface of ECs, resulting in the adhesion and migration of monocytes to the subendothelial space [36]. Adhesion molecules such as ICAM1, MMP9 and E-selectin have been suggested to be atherosclerotic inflammatory markers [4].

EP has been demonstrated to protect against the inflammatory response in some cardiovascular diseases. For example, it has the ability to inhibit neutrophil activation, inflammatory cytokine release, and nuclear factor κB translocation. EP has been associated with a delayed myocardial protective effect after regional ischemia and reperfusion (IR) injury in an in vivo rat heart model [37]. Liu and colleagues found that EP ameliorates monocrotaline-induced pulmonary arterial hypertension and reverses pulmonary vascular remolding in rats by inhibiting the release of TNF-α and IL-6 and by reducing the expression of endothelin-1 [38]. Additionally, EP has been shown to reduce the systemic inflammatory response and lung injury resulting from shock and IR in an experimental model of ruptured abdominal aortic aneurysm [39]. However, the protective effects and mechanisms underlying the action of EP against EC inflammatory injury have not been fully elucidated. In our study, exposure of HUVECs to TNF-α significantly increased their adhesion to U937 monocytes, while pretreatment with EP significantly inhibited the TNF-α-induced adhesion of U937 monocytes to HUVECs. In addition, exposure of HUVECs to TNF-α significantly increased sICAM1, IL-8, MCP-1 and sE-selectin production in the cell culture supernatants. TNF-α also significantly up-regulated ICAM-1 and MMP9 protein expression in HUVECs. In addition, EP treatment reversed the effects of TNF-α, and the effects were the most obvious at 10 mM EP.

Previous studies have confirmed that ERS plays an important role in regulating the pathological process of EC inflammation. Shinozaki and colleagues have confirmed that a deficiency in Herp (an ERS-related protein) suppresses atherosclerosis in apolipoprotein E knockout mice by attenuating the inflammatory response [21]. Remarkably, Zeng and colleagues have found that 4-phenylbutyric acid (an ERS inhibitor) suppresses inflammation by regulating the ERS in endothelial cells stimulated by uremic serum [23]. Furthermore, the pharmacological actions of other pyruvate derivatives are closely related to ERS. For example, 3-bromopyruvate induces ERS, overcomes autophagy and causes apoptosis in human hepatocellular carcinoma cell lines [24], [25]. Considering the above, we speculated that ERS may be involved in the anti-inflammatory effects of EP in ECs. In this study, TNF-α treatment significantly increased the ERS-related molecules in HUVECs (GRP78, ATF4, caspase12 and p-PERK). EP treatment effectively reversed the TNF-α-induced up-regulation of the ERS-related molecules and inflammatory injury. Furthermore, THA (an ERS inducer) not only up-regulated the ERS-related molecules (GRP78, ATF4, caspase12 and p-PERK) but also attenuated the protective effects of EP against TNF-α-induced inflammatory injury. PERK siRNA treatment not only inhibited the ERS-related molecules (GRP78, ATF4, caspase12 and p-PERK) but also mimicked the protective effects of EP in the attenuation of TNF-α-induced inflammatory injury.

In summary, we have demonstrated for the first time that EP can effectively reduce vascular endothelial inflammation and that this effect at least in part depends on the attenuation of ERS. Furthermore, the inhibition of the ERS pathway confers a protective effect against endothelial inflammatory injury, indicating that ERS is a crucial mediator of endothelial inflammation.
